# Draft Genome Sequence of *Prochlorococcus marinus* Strain XMU1401, Isolated from the Western Tropical North Pacific Ocean

**DOI:** 10.1128/genomeA.01431-17

**Published:** 2018-01-11

**Authors:** Wei Yan, Rui Zhang, Shuzhen Wei, Qinglu Zeng, Xilin Xiao, Qiong Wang, Hanrui Yan, Nianzhi Jiao

**Affiliations:** a State Key Laboratory of Marine Environmental Science, College of Ocean and Earth Sciences, Xiamen University, Xiamen, China; b Institute of Marine Microbes and Ecospheres, Xiamen University, Xiamen, China; c Division of Life Science, the Hong Kong University of Science and Technology, Clear Water Bay, Hong Kong, China

## Abstract

*Prochlorococcus marinus* is the most abundant photosynthetic organism in the tropical and subtropical oceans. Here, we report the draft genome sequence of *Prochlorococcus marinus* XMU1401, which was isolated from the Western Tropical North Pacific Ocean.

## GENOME ANNOUNCEMENT

The cyanobacterium *Prochlorococcus*, the smallest known oxygenic phototroph, dominates the tropical and subtropical oceans (1, 2) and can account for up to half of the net primary productivity (2). *Prochlorococcus* has a cluster of high-light (HL)-adapted ecotypes (HLI to HLVI) and a cluster of low-light (LL)-adapted ecotypes (LLI to LLVII) (3). The two most abundant ecotypes (HLI and HLII) have different distributions along the oceanic temperature gradient (4).

*Prochlorococcus marinus* strain XMU1401 was enriched and isolated from sea water collected from the Western Tropical North Pacific Ocean (WTNP) (18°N, 129°E). The initial isolation steps were performed as previously described (5), with the addition of stock solutions of Pro2 medium (6). XMU1401 was purified using a modified 96-well plate dilution-to-extinction method (7). The culture medium used for purification was ProMM, except an f/2 vitamin mix was used instead of the 1× Va vitamin mix (8). The culture was maintained in Pro99 medium (6) under constant light flux (10 μmol Q m^−2^ s^−1^) at 22°C.

Genomic DNA was collected from 25-ml cultures by centrifugation at 10,000 × *g* for 15 min, purified, and then used to construct an Illumina sequencing library. DNA libraries were prepared and sequenced on an Illumina HiSeq 2500 system at Shanghai Hanyu Biotechnology Co. (Shanghai, China). Raw sequencing reads were trimmed and quality controlled using Trimmomatic version 0.32 (9). High-quality paired-end reads (150 × 150 nucleotides [nt]) were assembled using Velvet version 1.2.03 (10).

The draft genome comprised nine contigs, with an *N*_50_ of 224,121 bp and a total length of 1,614,308 bp. The GC content was 31.3%. A total of 1,894 protein-coding sequences and 40 RNAs were predicted by the Rapid Annotations using Subsystems Technology (RAST) server (11). The total assembly size, GC content, and number of coding sequences are consistent with those of other sequenced HL strains (12). Phylogenetic analysis based on an internal transcribed spacer (ITS) revealed that XMU1401 is a strain of the HLII ecotype.

The WTNP is characterized by strong light radiation but low nutrient and primary production levels (13, 14), and its biogeochemical cycling has both local and global influences. Although it has a high abundance of *Prochlorococcus*, the WTNP is undersampled for this genus, because only a few strains have been isolated and sequenced from this region (12). Therefore, the isolation of this strain will facilitate more detailed and comparative studies of *Prochlorococcus* genomes in the WTNP.

### Accession number(s).

The entire genome sequence of this project has been deposited at GenBank under the accession number PESY00000000.
